# Molecular Analysis of Upper Tract and Bladder Urothelial Carcinoma: Results from a Microarray Comparison

**DOI:** 10.1371/journal.pone.0137141

**Published:** 2015-08-28

**Authors:** Thomas Sanford, Sima Porten, Maxwell V. Meng

**Affiliations:** Department of Urology, University of California San Francisco, San Francisco, California, United States of America; Southern Illinois University School of Medicine, UNITED STATES

## Abstract

**Introduction:**

Prior studies have shown genetic similarities between upper tract and bladder urothelial carcinoma. However, upper tract urothelial carcinoma tends to be higher grade than bladder urothelial carcinoma and tends to form in patients with certain familial conditions (e.g. Lynch Syndrome), indicating there may be unique biologic processes in these tumors. The purpose of this study was to evaluate the differences in gene expression between upper tract and bladder urothelial carcinoma using microarray data.

**Design, Setting, Participants:**

A search of publicly available microarray datasets identified a clinically annotated dataset of 12 upper tract and 20 bladder urothelial carcinoma specimens. Gene expression analysis of data derived from the Affymetrix HGU133Plus2 chip was performed. Bioconductor packages were used to evaluate clustering, differential gene expression, pathways relevant to oncology, and a basal/luminal signature in upper tract versus bladder urothelial carcinoma.

**Results:**

When separated by pathologic T stage, there was evidence of differential clustering among pT3 tumors and significant gene expression differences in 81 genes. Pathway analysis revealed differences in HGF and TNF signaling pathways. Upper tract tumors tended to have high expression of genes associated with a luminal subtype. One of the genes most highly expressed in upper tract tumors, SLITRK6, is the target of an antibody drug conjugate (AGS15E) currently in phase I clinical trials.

**Conclusions:**

This study provides evidence for molecular differences between upper tract and bladder urothelial carcinoma, some of which contribute to oncologic-relevant pathways. Upper tract tumors tended to express genes consistent with a luminal subtype. We also identify a marker, SLITRK6, as a potential target for patients with advanced upper tract urothelial carcinoma.

## Introduction

Urothelial carcinoma may arise at any location along the course of the urothelium between the urethra and the renal pelvis. Upper tract urothelial carcinoma (UTUC) is comprised of ureteral and renal pelvis carcinoma and accounts for only 5% of all urothelial carcinoma [1]. UTUC has been found to have similar chromosomal abnormalities and genetic mutations when compared with urothelial carcinoma of the bladder (UCB) [2]. However, there is evidence for unique pathogenic processes specific to UTUC. Patients with Hereditary Nonpolyposis Colorectal Cancer and Balkan Nephropathy have a higher incidence of UTUC [3,4]. UTUC has also been associated with anomalies in the activity of specific regulatory proteins [5,6]. The aim of this study was to evaluate the molecular differences between UTUC and UCB through analysis of gene expression data. In addition to finding significant molecular differences between upper tract and bladder urothelial carcinoma, upper tract tumors had a strong association with a luminal subtype and may have a higher tendency to express SLITRK6, a target of an antibody-drug complex currently in phase I trials.

## Material (Patients) and Methods

### Search for Datasets

Online repositories of genomic datasets including Gene Expression Omnibus and ArrayExpress were searched using the following terms: “upper tract”, “urothelial carcinoma”, “bladder carcinoma”, “ureter”, “ureteral”, and “renal pelvis.” Datasets were included only if the dataset had robust clinical annotation. Datasets without feature level RNA expression data were excluded. Raw data were downloaded in.CEL format and clinical data associated with each sample were abstracted. Clinical variables associated with UCB and UTUC specimens were compared with Fisher’s exact test for categorical variables and Wilcoxon rank-sum test for continuous variables. A p-value of <0.05 was considered significant.

### Normalization, Unsupervised, and Supervised Analysis

Data analysis was performed using BRB-ArrayTools (v 4.2.1) [7] and R v 3.1.1[8]. Raw data (.CEL) files were imported. The data were normalized using the Robust Multi-Array Average (RMA) algorithm[9]. Unsupervised analysis was performed using the Euclidian distance method of hierarchical clustering on all samples[10]. A separate hierarchical clustering analysis was performed for samples within each T stage. Supervised analysis was performed using a t-test with a random variance model to evaluate for differentially expressed genes with a p-value of <0.001. Differentially expressed genes from the class comparison analysis were individually evaluated using the *affy* package in R for potential insights into disease processes and novel markers[11].

### Pathway Analysis and Luminal versus Basal Signature

Pathway analysis was performed with the bioconductor package Parametric Gene Set Enrichment Analysis [12]. This package tested for increased or decreased activity of known oncogenic pathways in each sample. Normal human urothelial cells were used as a reference sample [13]. Using the R package *gplots*, a heatmap was created using the genes from the BASE47 signature, which has been shown to successfully differentiate tumors into basal or luminal subtypes [14]. All 20 UCB tumors and all 12 UTUC tumors were used in the creation of the basal versus luminal heatmap.

## Results

### Dataset Selection

One dataset from the Expression Project for Oncology met all inclusion criteria [15]. All.CEL files were downloaded from the Gene Expression Omnibus database (accession number GSE 2109). This dataset is a clinically annotated set of de-identified tumor samples from multiple institutions. The gene expression profile of all samples in this dataset was evaluated using the Affymetrix Human Genome HGU 133 Plus 2.0 Array. Within this dataset, there were a total of 32 samples with clinical annotation: 12 UTUC samples and 20 UCB samples. Clinical characteristics of the patients are listed in Table 1. There were significant differences between UTUC and UCB with respect to gender and for pT1 tumors but not for pT2/pT3/pT4 tumors and not for lymph node involvement or distant metastasis.

**Table 1:** Clinical characteristics.

	Upper Tract Urothelial Carcinoma (n = 12)		Bladder Urothelial Carcinoma (n = 20)		p-value
**Age Group (median)**					
** **	60–70		60–70		1.0
** **					
**Gender**					
Men	4 (33%)		16 (80%)		0.02
Women	8 (67%)		4 (20%)		
					
**Pathologic TNM stage**					
Ta	1	(8%)	0	(0%)	0.38
T1	4	(33%)	0	(0%)	0.01
T2	1	(8%)	14	(70%)	0.07
T3	6	(50%)	6	(30%)	0.28
T4	0	(0%)	0	(0%)	1.0
N+	2	(17%)	4	(20%)	1.0
M+	1	(8%)	0	(0%)	0.38
					
**Smoking (median pack years)**					
	36–40		36–40		1.0

### Data Analysis

**Fig 1:**
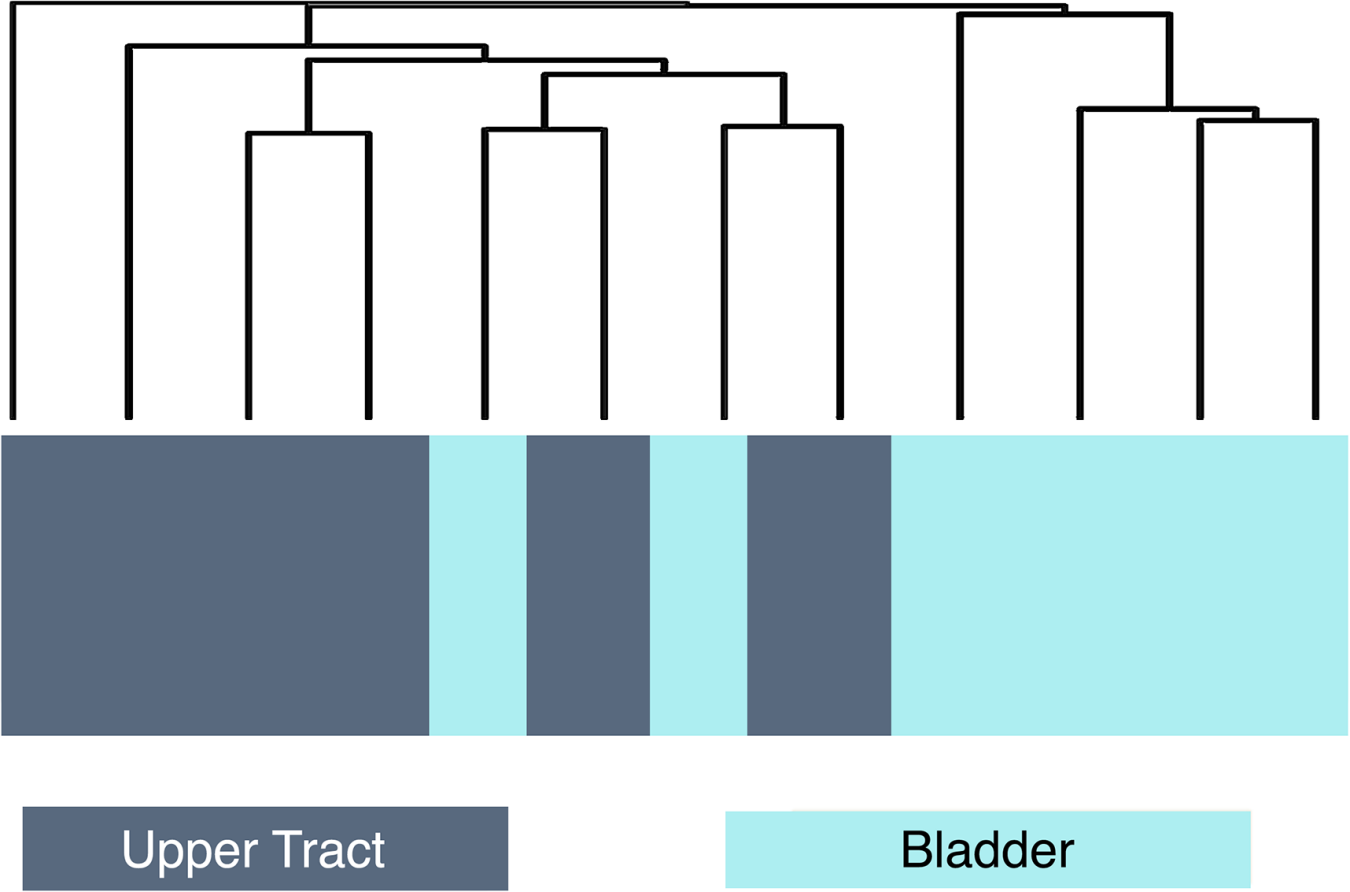
Hierarchical clustering of pT3 tumors shows differential clustering between upper tract and bladder urothelial carcinoma.

**Fig 2:**
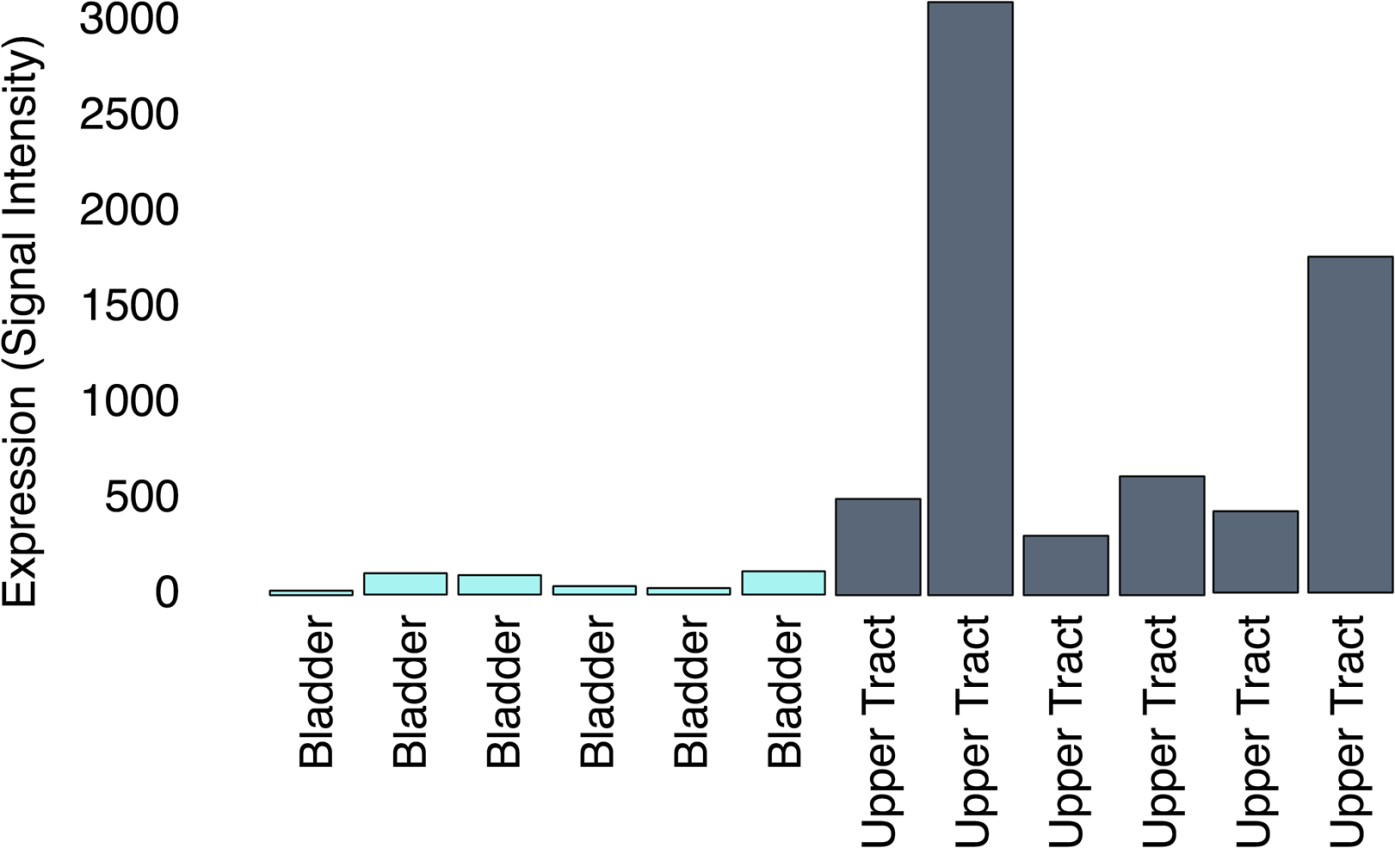
Higher levels of expression of SLITRK6 in UTUC than bladder UCB.

Hierarchical clustering of all samples did not reveal identifiable differences between UTUC and UCB (S1 Fig). However, when separated by pathologic T stage, there was evidence of differential clustering among pT3 tumors (Fig 1). Further class comparison analysis was performed using only pT3 tumors. There were a total of 81 genes differentially expressed between UTUC and UCB pT3 tumors (S2 Fig). The ten genes with the highest level of differential expression in UTUC and UCB are listed in Table 2. Genes over-expressed in UTUC were notably enriched for tyrosine kinase signaling and regulation of apoptosis. In addition, two are involved in immune and inflammatory pathways (IFI27 and HCP5). One gene found to be over-expressed in upper tract urothelial carcinoma, SLITRK6, is an integral membrane protein known to have high levels of expression in certain carcinomas but low levels of expression in most other tissues [16]. This marker has been shown to have high levels of expression in UCB[17]. The analysis in this study compares expression of UTUC to UCB and therefore upper tract urothelial carcinoma appears to have particularly high levels of expression of this gene (Fig 2).

**Table 2:** Top Over-Expressed Genes in Bladder and Upper Tract Urothelial Carcinoma.

Over-expressed in Bladder Urothelial Carcinoma	Over-expressed in Upper Tract Urothelial Carcinoma
(IFI27) Interferon alpha inducible protein 27	(SLITRK6) SLIT and NTRK-like family, member 6
(ACTG2) Actin, Gamma 2	(TSPAN12) Tetraspanin 12
(HCP5) HLA complex P5	(PLS1) Plastin 1
(ALDH1A3) Aldehyde dehydrogenase 1 family, A3	(SPGL1) Spingosine-1-phsphate lyase 1
(MCL1) Myeloid cell leukemia sequence 1	(HOXB4) Homeobox B4

### Pathway Analysis and Luminal versus Basal Signature

**Fig 3:**
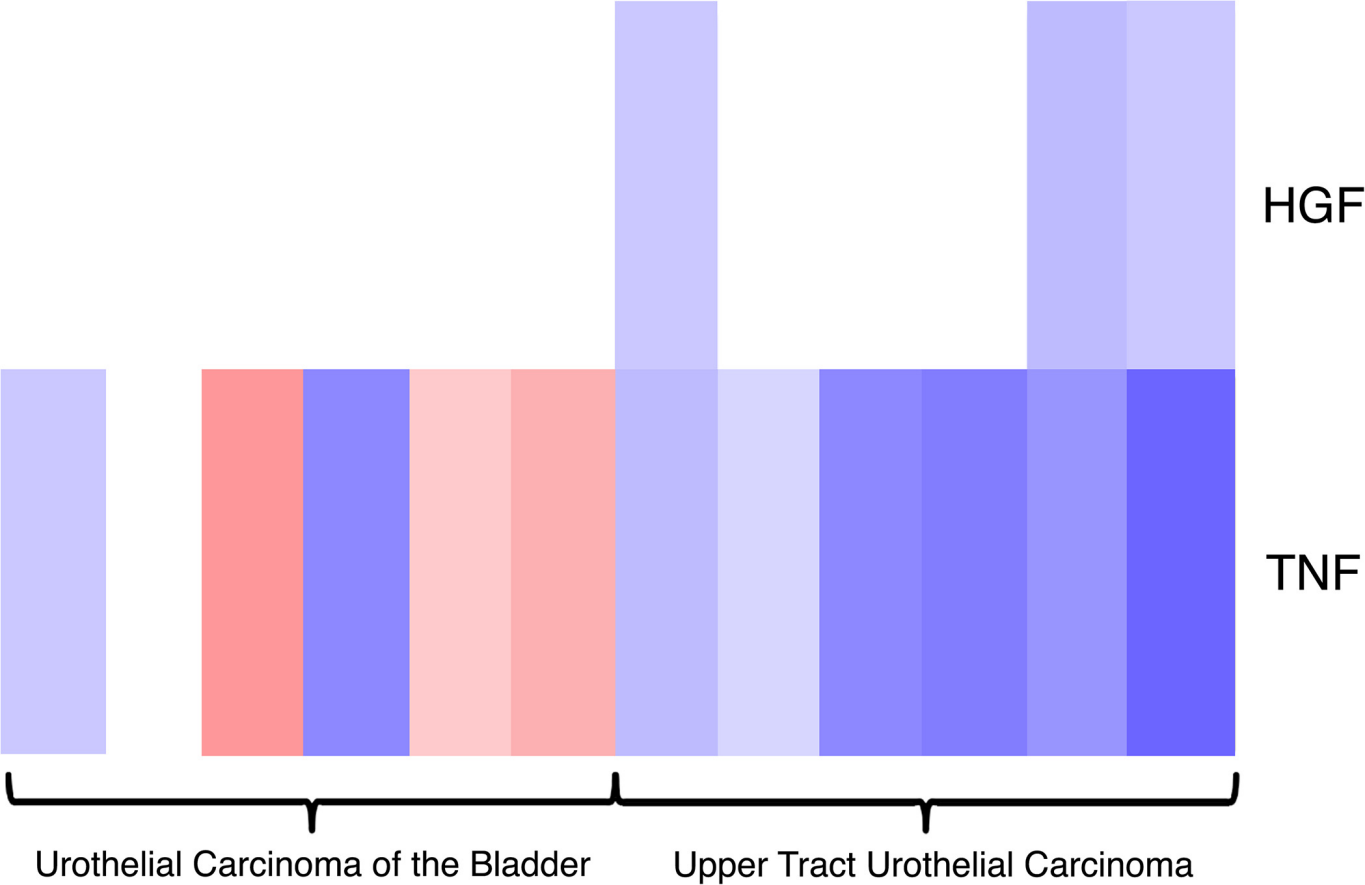
Pathway analysis shows low expression of HGF and TNF in upper tract urothelial carcinoma. Blue indicates lower expression and red indicates higher expression.

**Fig 4:**
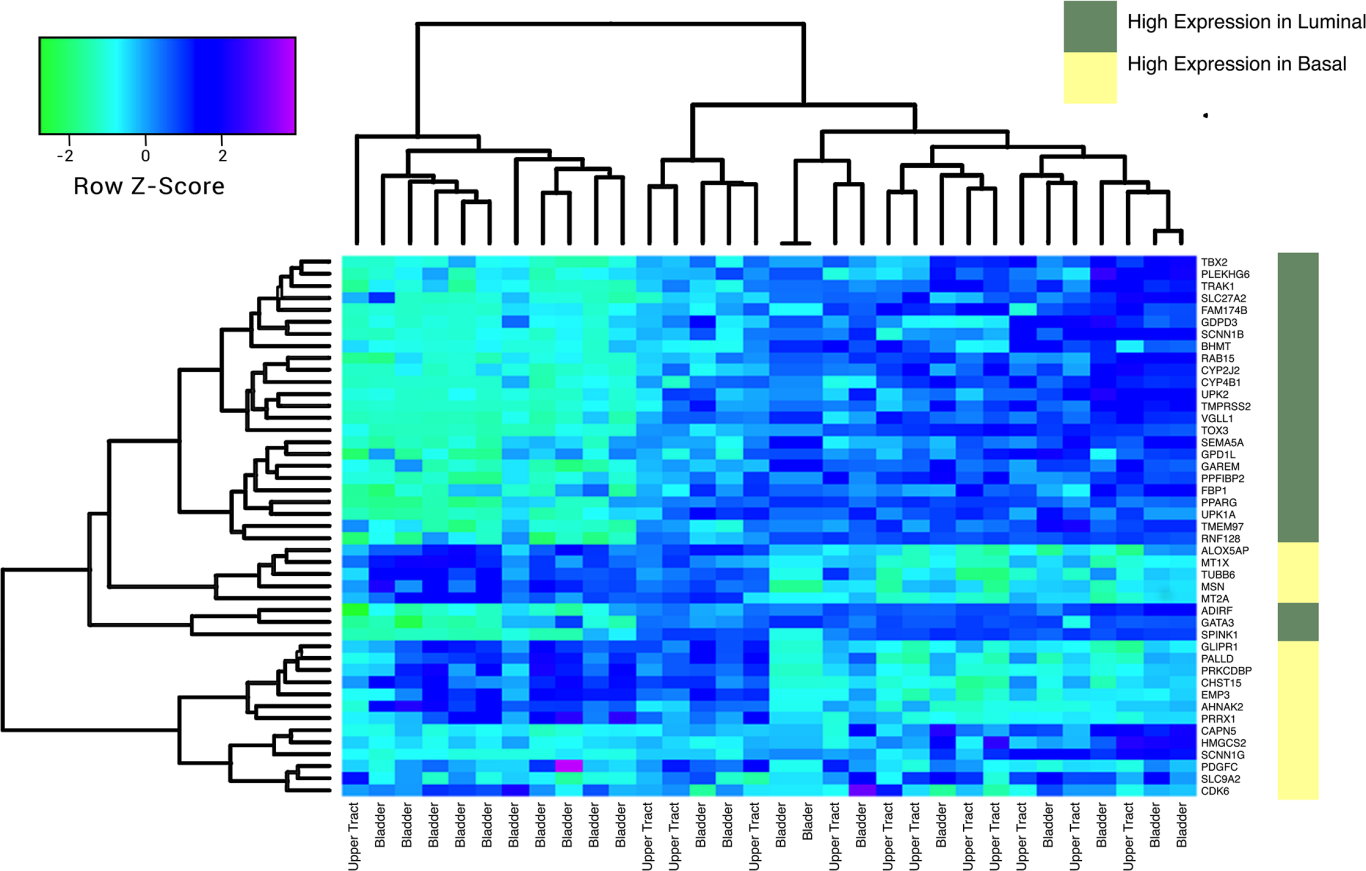
Heatmap of BASE47 gene signature with high expression of luminal type markers in UTUC.

Pathway analysis using the R package Parametric Gene Set Enrichment Analysis found under-expression in the TNF and HGF pathways in UCB compared with UTUC (Fig 3). When hierarchical clustering was performed using the BASE47 gene signature, upper tract tumors tended to cluster in a group with high expression of luminal genes (Fig 4).

## Discussion

UTUC is an uncommon malignancy with an incidence of 0.7/100,000 person years[18]. Definitive treatment consists of radical nephroureterectomy with resection of the intramural ureter. The most important prognostic factor following surgical resection is pathologic tumor stage: the 5 year survival is 88–91% in superficial disease (Tis, Ta, T1), 71% in T2 disease, 48% in T3 disease, and <5% for T4 disease[19]. Unlike UCB in which approximately 75% of cases are superficial at the time of diagnosis, 45% of UTUC are invasive when diagnosed [20]. The disease-specific survival has improved slightly over the past 50 years, but overall 5-year disease-specific mortality remains at 25%, reflecting the tendency for advanced stage at diagnosis [21]. Currently, patients with metastatic or locally advanced UTUC are treated with chemotherapy regimens that have proven effective in patients with locally advanced or metastatic urothelial carcinoma derived from the bladder: MVAC (methotrexate, vinblastine, doxorubicin, cisplatin) or a combination of gemcitabine and cisplatin. However, there are no prospective trials showing survival benefit for these agents in patients with advanced UTUC, and renal insufficiency after extirpative surgery may limit chemotherapeutic treatment options [22,23]. This study sought to evaluate genetic anomalies specific to UTUC that may assist in our understanding of the disease as well as identify possible prognostic or therapeutic markers.

Prior studies evaluating the molecular biology of UCB and UTUC have focused on genetic similarities. On the chromosomal level, many of the same genetic abnormalities found in UCB are also observed in UTUC [24]. Mutations in specific genes including FGFR3, p53, and Rb have been associated with both UTUC and UCB [25,26]. However, microscopic evaluation has shown that UTUC is generally more aggressive (i.e. higher grade) than UCB [1]. Furthermore, there are specific environmental exposures and hereditary syndromes associated with an increased risk of development of UTUC. Patients with Hereditary Nonpolyposis Colorectal Cancer have a defect in DNA repair genes resulting in a hypermutable state. These patients are known to have a 22 fold increased risk of developing UTUC [27]. Furthermore, individuals in the Balkan Peninsula are at risk for developing Balkan Endemic Nephropathy, which is a chronic renal disease that produces interstitial fibrosis, renal insufficiency, and an increased risk of UTUC [3]. The increase in incidence of UTUC in these specific patient populations raises the possibility that specific pathways are activated or suppressed in UTUC.

Prior studies using high throughput genomic analysis of UTUC have provided insightful findings. Zhang et al performed a study using gene expression microarrays to compare UTUC to muscle invasive UCB. Although similar gene expression profiles were found when comparing UCB and UTUC in unsupervised analysis, differences in expression of sodium ion transporters were found in class comparison analysis [28]. Quian et al found a high level of activity in the AKT/PI3K pathway in UTUC. This group went on to create a mouse model with a PTEN deletion which resulted in increased AKT/mTOR signaling in upper tract urothelial carcinoma [29]. Patel et al reviewed multiple genetic studies of UTUC and proposed a model by which deficiencies in DNA repair, as seen in disease states that increase the risk of UTUC (i.e. Lynch syndrome), leads to activation of the AKT/mTOR pathway and subsequent development of UTUC[25].

Our data show the gene expression profiles between UTUC and UCB were very similar until separated by pathologic T stage. This finding may be explained by the molecular heterogeneity in urothelial carcinoma. The recently published Cancer Genome Atlas Research Network study of muscle invasive UCB showed recurrent mutations in 32 unique genes as well as a wide variety of copy number alterations, confirming that muscle invasive UCB is indeed a molecularly heterogeneous disease[30]. Furthermore, urothelial carcinoma has been shown to have an increased number of genetic abnormalities as stage and grade increases[31]. Integration of stage and molecular information may be necessary to detect subsets of urothelial carcinoma with specific genetic changes.

Our supervised analysis was based on a subset of pT3 tumors and showed differential expression of 81 genes. Many of the genes that were overexpressed in UCB were immunologic mediators such as HLA complex P5 and IFI27. This is an interesting finding given the critical role immunotherapy such as Bacillus Calmette-Guerin in treating non-muscle invasive bladder cancer, as well as the current activity in elucidating the role of PD-L1/PD-1 pathway in UCB[32].

Pathway analysis showed lower levels of expression of genes enriched in the TNF pathway and the HGF pathway in UTUC compared with UCB. This may have implications in the response to immune mediated therapies, such as BCG and interferon [22]. We also found over-expression of a marker with potential therapeutic value, SLITRK6. This is a membrane protein with low levels of expression in normal tissues, but high levels of expression in certain types of cancer including UCB, making it an ideal target for an antibody-drug therapeutic agent[16]. An antibody to the SLITRK6 protein has been linked with a cytotoxic agent monomethyl auristatin E (AGS15E) and is currently in phase I clinical trials for patients with metatstatic urothelial carcinoma (protocol ID NCT 01963052)[17]. Given our analysis compared the relative expression of UCB and UTUC and this gene was found to be over-expressed in UTUC relative to UCB, AGS15E may be a good drug candidate for patients with locally advanced or metastatic UTUC who otherwise have limited treatment options.

Genes associated with a luminal subtype were highly expressed in UTUC. The basal versus luminal subtypes are thought to represent different stages of differentiation[14]. This finding lends further support to the idea that UTUC represents a unique subpopulation of urothelial carcinoma.

This study has a number of limitations. First, the numbers are small given the rarity of the disease. Second, this study was conducted using publically available data and there was no central review of the pathology. Despite these limitations, we believe this is the first study of gene expression comparing UTUC and UCB to find specific examples that upper tract tumors may represent a unique sub-population of urothelial carcinoma.

## Conclusion

By separating upper tract urothelial carcinoma and bladder urothelial carcinoma by T stage in our analysis of microarray data, we were able to detect molecular differences in UTUC when compared to UCB. Differentially expressed genes have immunogenic functions, and were involved in the TNF and HFG pathways. Upper tract tumors tend to over-express luminal genes. One gene highly over-expressed in upper tract urothelial carcinoma, SLITRK6, is the target of an antibody-drug conjugate currently being evaluated in phase I clinical trials.

## Supporting Information

S1 Fig Hierarchical clustering of tumors of all stages shows no differential clustering between UTUC and UCB. (TIF)

S2 Fig Differential clustering between UTUC (right side) and UCB (left side) in pT3 tumors. (TIF)

## References

[1] Stewart GD, Bariol SV, Grigor KM, Tolley DA, McNeill SA. A comparison of the pathology of transitional cell carcinoma of the bladder and upper urinary tract. BJU Int. 2005;95: 791–793. 15794784 10.1111/j.1464-410X.2005.05402.x

[2] Bing Z, Li J, Master SR, Lee C-C, Puthiyaveettil R, Tomaszewski JE. Fluorescence in situ hybridization study of chromosome abnormalities of upper urinary tract urothelial carcinoma in paraffin-embedded tissue. Am J Clin Pathol. 2012;138: 382–389. 10.1309/AJCPUXAP6P2GVBTI 22912355

[3] Stefanovic V, Toncheva D, Atanasova S, Polenakovic M. Etiology of Balkan endemic nephropathy and associated urothelial cancer. Am J Nephrol. 2006;26: 1–11. 16391464 10.1159/000090705

[4] Rouprêt M, Yates DR, Comperat E, Cussenot O. Upper urinary tract urothelial cell carcinomas and other urological malignancies involved in the hereditary nonpolyposis colorectal cancer (lynch syndrome) tumor spectrum. Eur Urol. 2008;54: 1226–1236. 10.1016/j.eururo.2008.08.008 18715695

[5] Nakanishi K, Ogata S, Matsuo H, Kanai Y, Endou H, Hiroi S, et al Expression of LAT1 predicts risk of progression of transitional cell carcinoma of the upper urinary tract. Virchows Arch. 2007;451: 681–690. 17622555 10.1007/s00428-007-0457-9

[6] Uematsu K, Ogata S, Nakanishi K, Hiroi S, Tominaga S, Aida S, et al Glucose-regulated protein 78 expression in urothelial carcinoma of the upper urinary tract. BJU Int. 2010;106: 873–878. 10.1111/j.1464-410X.2009.09144.x 20039870

[7] Simon R, Lam A, Li M-C, Ngan M, Menenzes S, Zhao Y. Analysis of gene expression data using BRB-ArrayTools. Cancer Inform. 2007;3: 11–17. 19455231 PMC2675854

[8] Ihaka R, Gentleman R. R: A Language for Data Analysis and Graphics. Journal of Computational and Graphical Statistics of computational and graphical statistics; 1996;5: 299–314. Available: http://biostat.mc.vanderbilt.edu/wiki/pub/Main/JeffreyHorner/JCGSR.pdf

[9] Irizarry RA, Hobbs B, Collin F, Beazer-Barclay YD, Antonellis KJ, Scherf U, et al Exploration, normalization, and summaries of high density oligonucleotide array probe level data. Biostatistics. 2003;4: 249–264. 12925520 10.1093/biostatistics/4.2.249

[10] Do JH, Choi D-K. Clustering approaches to identifying gene expression patterns from DNA microarray data. Mol Cells. 2008;25: 279–288. 18414008

[11] Gautier L, Cope L, Bolstad BM, Irizarry RA. affy—analysis of Affymetrix GeneChip data at the probe level. Bioinformatics. 2004;20: 307–315. 14960456 10.1093/bioinformatics/btg405

[12] Furge KA, Chen J, Koeman J, Swiatek P, Dykema K, Lucin K, et al Detection of DNA copy number changes and oncogenic signaling abnormalities from gene expression data reveals MYC activation in high-grade papillary renal cell carcinoma. Cancer Res. 2007;67: 3171–3176. 17409424 10.1158/0008-5472.CAN-06-4571

[13] Erickson DR, Schwarze SR, Dixon JK, Clark CJ, Hersh MA. Differentiation associated changes in gene expression profiles of interstitial cystitis and control urothelial cells. J Urol. 2008;180: 2681–2687. 10.1016/j.juro.2008.08.007 18951569

[14] Damrauer JS, Hoadley KA, Chism DD, Fan C, Tiganelli CJ, Wobker SE, et al Intrinsic subtypes of high-grade bladder cancer reflect the hallmarks of breast cancer biology. Proc Natl Acad Sci. 2014;111: 3110–3115. 10.1073/pnas.1318376111 24520177 PMC3939870

[15] Expression Project for Oncology [Internet]. [cited 27 Jan 2015]. Available: http://www.intgen.org/expo/

[16] Aruga J, Yokota N, Mikoshiba K. Human SLITRK family genes: genomic organization and expression profiling in normal brain and brain tumor tissue. Gene. 2003;315: 87–94. 14557068 10.1016/s0378-1119(03)00715-7

[17] Morrison K. Development of AGS15E: a Novel Antibody Drug Conjugate Targeting SLITRK6 for the Treatment of Bladder Cancer. 2012. Available: http://adc-summit.com/uploads/files/1972/Kendall_Morrison.pdf

[18] Munoz JJ, Ellison LM. Upper tract urothelial neoplasms: incidence and survival during the last 2 decades. J Urol. 2000;164: 1523–1525. 11025695

[19] Margulis V, Shariat SF, Matin SF, Kamat AM, Zigeuner R, Kikuchi E, et al Outcomes of radical nephroureterectomy: a series from the Upper Tract Urothelial Carcinoma Collaboration. Cancer. 2009;115: 1224–1233. 10.1002/cncr.24135 19156917

[20] Olgac S, Mazumdar M, Dalbagni G, Reuter VE. Urothelial carcinoma of the renal pelvis: a clinicopathologic study of 130 cases. Am J Surg Pathol. 2004;28: 1545–1552. 15577672 10.1097/00000478-200412000-00001

[21] Abouassaly R, Alibhai SMH, Shah N, Timilshina N, Fleshner N, Finelli A. Troubling outcomes from population-level analysis of surgery for upper tract urothelial carcinoma. Urology. 2010;76: 895–901. 10.1016/j.urology.2010.04.020 20646743

[22] Audenet F, Yates DR, Cussenot O, Rouprêt M. The role of chemotherapy in the treatment of urothelial cell carcinoma of the upper urinary tract (UUT-UCC). Urol Oncol. 2013;31: 407–413. 10.1016/j.urolonc.2010.07.016 20884249

[23] Ouzzane A, Colin P, Xylinas E, Pignot G, Ariane MM, Saint F, et al Ureteral and multifocal tumours have worse prognosis than renal pelvic tumours in urothelial carcinoma of the upper urinary tract treated by nephroureterectomy. Eur Urol. 2011;60: 1258–1265. 10.1016/j.eururo.2011.05.049 21665356

[24] Rigola MA, Fuster C, Casadevall C, Bernués M, Caballín MR, Gelabert A, et al Comparative genomic hybridization analysis of transitional cell carcinomas of the renal pelvis. Cancer Genet Cytogenet. 2001;127: 59–63. 11408067 10.1016/s0165-4608(00)00426-x

[25] Patel N, Arya M, Muneer A, Powles T, Sullivan M, Hines J, et al Molecular aspects of upper tract urothelial carcinoma. Urol Oncol. 2014;32: 28.e11–20. 10.1016/j.urolonc.2012.10.002 23428541

[26] Chan P-H, Kho VK-S, Lai S-K, Yang C-H, Chang H-C, Chiu B, et al Treatment of emphysematous pyelonephritis with broad-spectrum antibacterials and percutaneous renal drainage: an analysis of 10 patients. J Chin Med Assoc. 2005;68: 29–32. 15742860 10.1016/S1726-4901(09)70128-5

[27] Watson P, Lynch HT. The tumor spectrum in HNPCC. Anticancer Res. 1994;14: 1635–1639. 7979199

[28] Zhang Z, Furge KA, Yang XJ, Teh BT, Hansel DE. Comparative gene expression profiling analysis of urothelial carcinoma of the renal pelvis and bladder. BMC Med Genomics. 2010;3: 58 10.1186/1755-8794-3-58 21159190 PMC3022544

[29] Qian C-N, Furge KA, Knol J, Huang D, Chen J, Dykema KJ, et al Activation of the PI3K/AKT pathway induces urothelial carcinoma of the renal pelvis: identification in human tumors and confirmation in animal models. Cancer Res. 2009;69: 8256–8264. 10.1158/0008-5472.CAN-09-1689 19843858 PMC2783739

[30] Cancer Genome Atlas Research Network. Comprehensive molecular characterization of urothelial bladder carcinoma. Nature. 2014;507: 315–322. 10.1038/nature12965 24476821 PMC3962515

[31] Blaveri E. Bladder cancer stage and outcome by array-based comparative genomic hybridization. Clin Cancer Res. 2005;11: 7012–7022. 16203795 10.1158/1078-0432.CCR-05-0177

[32] Carneiro BA, Meeks JJ, Kuzel TM, Scaranti M, Abdulkadir SA, Giles FJ. Emerging therapeutic targets in bladder cancer. Cancer Treat Rev. 2015;41: 170–178. 10.1016/j.ctrv.2014.11.003 25498841

