# Supplementary figures and images for: Molecular Analysis of Upper Tract and Bladder Urothelial Carcinoma: Results from a Microarray Comparison
Source: PLoS One. 2015 Aug 28;10(8):e0137141. doi: 10.1371/journal.pone.0137141 (PMC4552875; doi:10.1371/journal.pone.0137141)

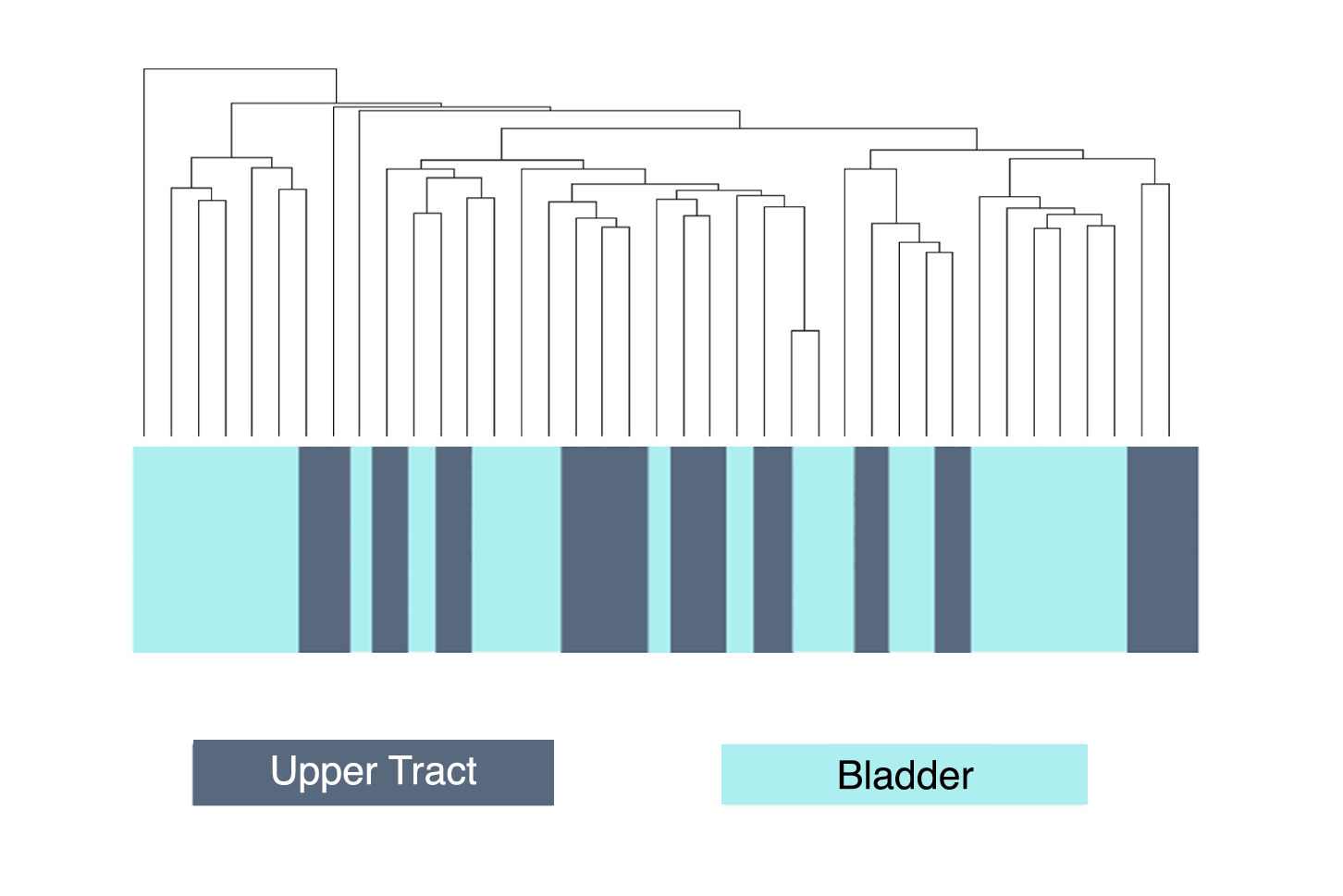

Supplement: S1 Fig — (TIF) [file pone.0137141.s001.tif]

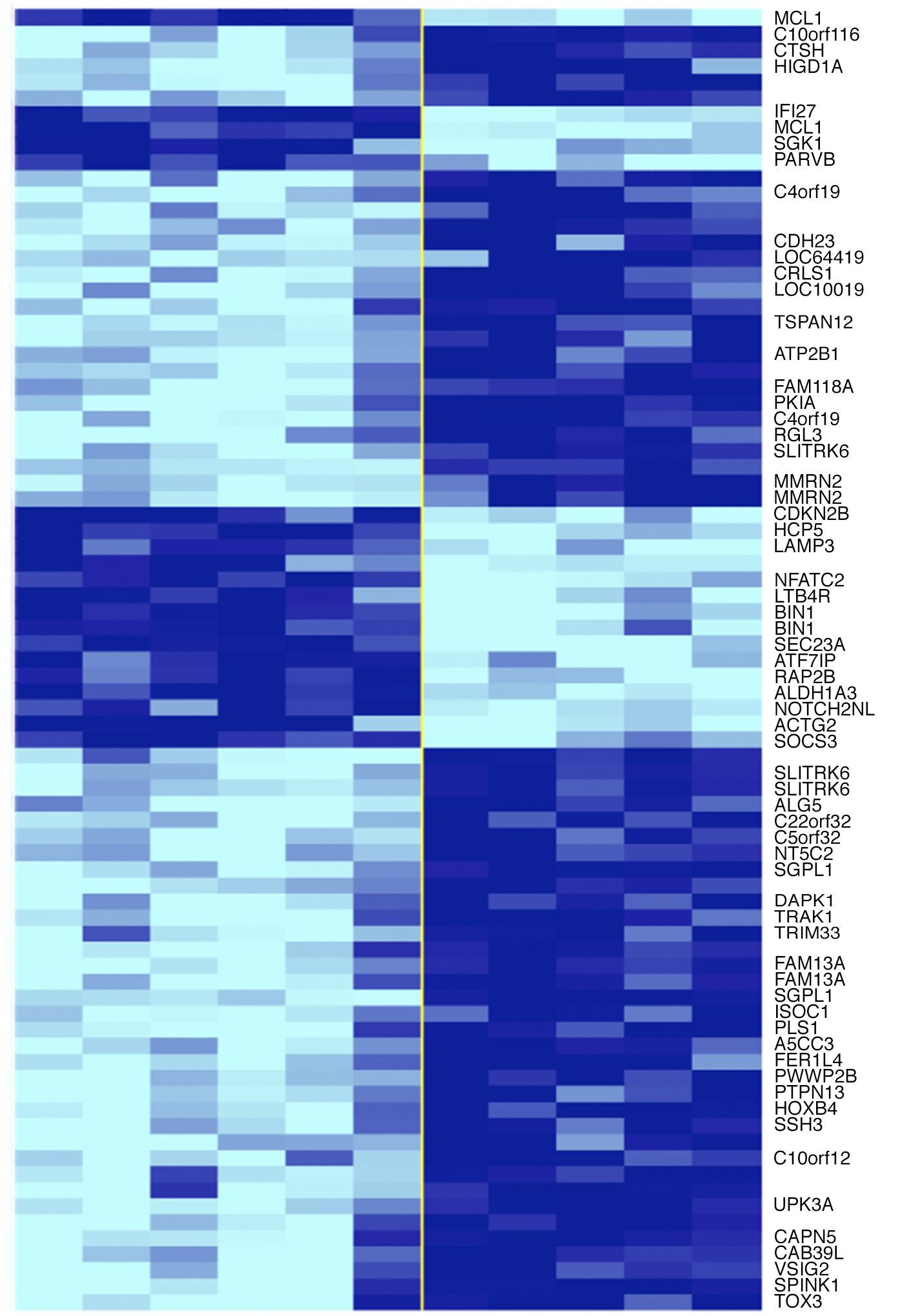

Supplement: S2 Fig — (TIF) [file pone.0137141.s002.tif]
